# mRNA Display Selection of an Optimized MDM2-Binding Peptide That Potently Inhibits MDM2-p53 Interaction

**DOI:** 10.1371/journal.pone.0017898

**Published:** 2011-03-15

**Authors:** Hirokazu Shiheido, Hideaki Takashima, Nobuhide Doi, Hiroshi Yanagawa

**Affiliations:** Department of Biosciences and Informatics, Keio University, Yokohama, Japan; Leeds Institute of Molecular Medicine, United Kingdom

## Abstract

p53 is a tumor suppressor protein that prevents tumorigenesis through cell cycle arrest or apoptosis of cells in response to cellular stress such as DNA damage. Because the oncoprotein MDM2 interacts with p53 and inhibits its activity, MDM2-p53 interaction has been a major target for the development of anticancer drugs. While previous studies have used phage display to identify peptides (such as DI) that inhibit the MDM2-p53 interaction, these peptides were not sufficiently optimized because the size of the phage-displayed random peptide libraries did not cover all of the possible sequences. In this study, we performed selection of MDM2-binding peptides from large random peptide libraries in two stages using mRNA display. We identified an optimal peptide named MIP that inhibited the MDM2-p53 and MDMX-p53 interactions 29- and 13-fold more effectively than DI, respectively. Expression of MIP fused to the thioredoxin scaffold protein in living cells by adenovirus caused stabilization of p53 through its interaction with MDM2, resulting in activation of the p53 pathway. Furthermore, expression of MIP also inhibited tumor cell proliferation in a p53-dependent manner more potently than DI. These results show that two-stage, mRNA-displayed peptide selection is useful for the rapid identification of potent peptides that target oncoproteins.

## Introduction

p53 is a tumor suppressor protein that prevents tumorigenesis [1], [2]. By responding to cellular stress such as DNA damage, expression levels of p53 increase, and the upregulated p53 transactivates various targets involved in antitumor activities such as cyclin-dependent kinase inhibitor p21^WAF1/CIP1^ and the pro-apoptotic protein Puma [3], [4]. Consequently, p53 induces cell cycle arrest or apoptosis in cells that have genetic aberrations, and as such, inactivation of p53 leads to accumulation of the aberrations that may cause overexpression of several kinds of oncoproteins, resulting in tumorigenesis [5]. p53 retains its wild-type status in approximately 50% of human cancers. Therefore, inactivation of p53 is caused by interaction with the E3 ubiquitin ligase MDM2 [6]–[8]. MDM2 acts as an essential regulator of p53 stability and activity by forming a negative feedback loop [9]. Several studies have shown that abrogating the MDM2-p53 interaction leads to reactivation of the p53 pathway and inhibition of tumor cell proliferation [10], [11]. The crystal structure of the MDM2-p53 complex revealed that the N-terminal portion of p53_15–29_ is important in binding to MDM2, and several small-molecule compounds or peptides mimicking the MDM2 binding site of p53 antagonize MDM2 and activate the p53 pathway in cancer cells [12]–[15]. Therefore, the MDM2-p53 interaction is a potent target of anticancer drug design [16], [17].

Peptides are powerful tools for disrupting protein-protein interactions because the large interacting surfaces and the high specificity of these peptides lead to fewer adverse side effects when used as pharmaceutical agents [18], [19]. As previously reported, several peptides that inhibit the MDM2-p53 interaction have been identified from randomized peptide libraries using phage display [20], [21]. Hu et al. identified a 12-amino-acid (aa) peptide (LTFEHYWAQLTS), DI, that could inhibit not only the MDM2-p53 interaction but also the MDMX-p53 interaction more effectively than Nutlin-3, a small molecular inhibitor of the MDM2-p53 interaction [10], [12]. An MDM2 homologue, MDMX is highly expressed in tumors which also binds to and negatively regulates p53 [12]. Furthermore, DI expressed with recombinant adenovirus as a thioredoxin-fused protein could activate the p53 pathway both *in vitro* and *in vivo*. However, DI was not sufficiently optimized because it was selected by phage display from a 12-mer random library (4.1×10^15^ possible members) with a size of ∼10^8^ that did not cover all of the possible sequences.

To overcome this problem, we performed *in vitro* selection of MDM2-binding peptides from random peptide libraries using mRNA display [22], [23]. This system based on cell-free translation is a potent method for screening large peptide libraries (∼10^13^ unique members) that can cover all of the possible sequences in a 10-mer random library. In this study, we applied mRNA display to identify a highly optimized peptide that could disrupt the MDM2-p53 complex from a random library containing all of the possible sequences by dividing the selection process into two stages. We also verified that a selected peptide could inhibit the MDM2-p53 interaction in living cells and block tumor cell growth.

## Results

### The 1st selection of a 16-mer randomized peptide library

**Figure 1:**
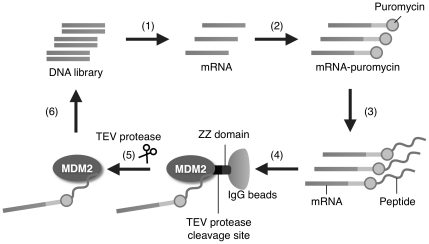
Schematic representation of *in vitro* selection of MDM2-binding peptides using mRNA display. (1) A DNA library encoding randomized peptides is transcribed. (2) The resulting RNA library is ligated with a PEG-Puro spacer and (3) *in vitro* translated to form a peptide-mRNA conjugate library. (4) The mRNA-displayed peptide library is incubated with MDM2 immobilized on beads through an affinity selection tag containing a ZZ domain and a TEV protease cleavage site [25], and unbound molecules are washed away. (5) The bound molecules are eluted by cleavage with the TEV protease, and (6) their mRNA portion is amplified by RT-PCR. The resulting DNA can be used for the next rounds of selection or analyzed by cloning and sequencing.

**Figure 2:**
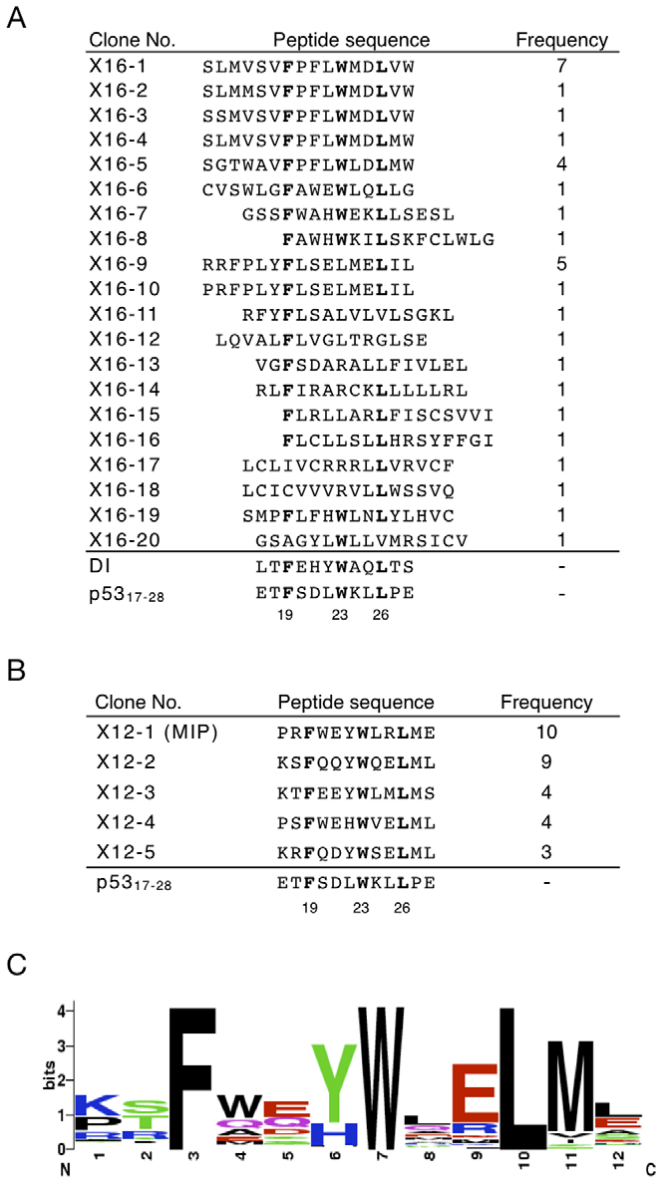
A multiple-sequence alignment of the selected peptides. Peptide sequences selected from randomized mRNA-displayed peptide libraries were aligned using the ClustalW program. The peptide sequences are shown using the single-letter code. (A) 16-mer peptides selected after four rounds of selection. Three amino acid residues, Phe, Trp and Leu, which were conserved in nearly every peptide, are shown in bold. (B) 12-mer peptides after five rounds of selection. Fixed amino acid residues are shown in bold. Values below the displayed p53_17–28_ sequence indicate the position of amino acids in the p53 protein. (C) Sequence logos representations were created with webLogo version 2.8.2 (http://weblogo.berkeley.edu/) based on the 83 peptide sequences obtained using the mRNA display system to select peptides that bound to MDM2 from the 12-mer partially randomized peptide library. The height of each column reflects the bias of particular residues. Polar amino acids containing an amide group and the amino acids that do not contain an amide group are shown in purple and green, respectively. Acidic and basic charged residues are shown in red and blue, respectively, while the hydrophobic residues are shown in black.

To obtain novel peptides capable of disrupting the MDM2-p53 complex using mRNA display (Fig. 1), we first constructed a 16-mer randomized peptide library encoded by (NNS)_16_ codons (N  =  A, T, G or C; S  =  G or C) because the crystal structure of the MDM2-p53 complex has revealed that the 15-aa residue of p53_15–29_ is important for binding to MDM2 [24]. As the bait protein, we used MDM2_7–300_ fused to the TAP tag [25], which contains the IgG binding domain of protein A (ZZ domain), a TEV protease cleavage site and a calmodulin binding peptide for immobilization on IgG beads and specific elution of the MDM2-binding peptide from beads during the affinity selection. After four rounds of selection for binding to the beads immobilizing the TAP-tagged MDM2, the resulting library was cloned and sequenced. Consequently, 33 peptide sequences were identified (Fig. 2A). More than half of all peptides retained the three hydrophobic residues corresponding to Phe19, Trp23, and Leu26 of wild-type p53. Three of the 33 peptides, X16-1, X16-5 and X16-9, were frequently obtained. Furthermore, DNA sequences of clones X16-1 and X16-9 were quite similar to those of X16-2 to X16-4 and X16-10, respectively, suggesting that these peptides were generated from acquired point mutations during RT-PCR in the selection.

To examine the ability of the peptides to bind to MDM2, we constructed plasmids for the expression of GFP-fused peptides and performed pull-down assays between the GFP-fused peptides and MDM2 (Fig. S1). Consequently, binding to MDM2 immobilized on beads was clearly detected for GFP-X16-1, X16-2, X16-5 and X16-9 but not for GFP-p53_13–28_ and GFP alone, suggesting that the affinity of the 16-mer peptides obtained from the selection was higher than that of wild-type p53. As previously reported, inhibition of the MDM2-p53 interaction leads to activation of the p53 pathway [10], [13], [14]. However, expression of these GFP-fused 16-mer peptides did not activate the p53 pathway in living cells (data not shown), suggesting that the ability of these peptides to bind to MDM2 or inhibit the MDM2-p53 interaction was insufficient.

### The 2nd selection of a 12-mer partially randomized peptide library

While the mRNA display selection system could select 10^12^–10^13^ molecules at once, it could cover at most 10^−7^–10^−8^ of the possible sequences in the 16-mer randomized library (∼10^20^). Therefore, these 16-mer peptides required further optimization. To improve the efficiency of the selection strategy, we performed a 2nd selection. The results from the prior selection suggest that three hydrophobic residues corresponding to Phe19, Trp23, and Leu26 of wild-type p53 are quite important in binding to MDM2, consistent with previous reports [24]. Additionally, on the basis of the findings that the 12-mer peptides obtained by phage display could tightly bind to MDM2 [12], [20], [21], we speculated that peptides of 12 aa residues were sufficient to antagonize MDM2 function. Thus, we constructed a 12-mer partially randomized library containing the three fixed hydrophobic residues encoded by the (NNS)_2_-TTC-(NNS)_3_-TGG-(NNS)_2_-TTA-(NNS)_2_ codons whose 5.1×10^11^ possible members were covered by the library size of mRNA display.

**Figure 3:**
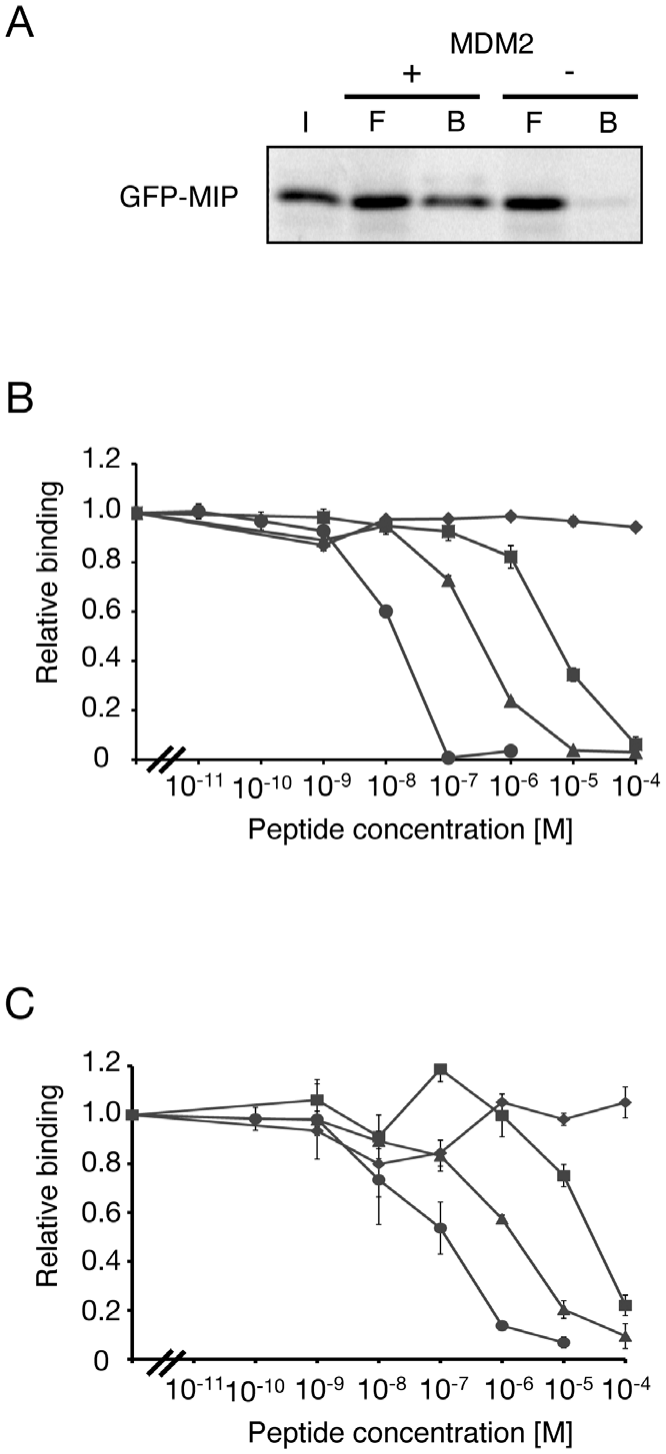
Inhibition of MDM2-p53 and MDMX-p53 interactions by synthetic peptides. (A) GFP-tagged MIP was generated by a transcription/translation reaction and used for *in vitro* binding assays as described in the “Materials and Methods” section. I, input; F, flow-through; B, beads. (B) MDM2 or (C) MDMX was generated by an *in vitro* transcription reaction, bound to His_6_-p53 immobilized on copper-coated plates in the presence of various concentrations of synthetic MIP (circle), DI (triangle), 3A (diamond) or p53 (square) peptides and quantified by ELISA. The IC_50_ values are shown in Table 1.

During the 2nd selection step, we found that enrichment of specific peptide sequences was difficult due to an abundance of molecules that bound to MDM2 in the library. This difficulty arose from the fixed key residues and the large library size of mRNA display. We therefore increased the stringency of the selection process to enrich for specific peptide sequences during the 2nd selection by shortening the binding time from 2 h to 5 min and increasing the number of washes from 3 to 15. After five rounds of the improved affinity selection, the amounts of the mRNA-displayed peptides that bound to the TAP-tagged MDM2 were saturated, and the resulting library was subsequently cloned and sequenced. The results show that sequences of 10 of the 83 peptides obtained from the library after the fifth rounds of selection were identical and were obtained most frequently (Fig. 2B). Therefore, the peptide X12-1 was identified as an optimized peptide for disrupting the MDM2-p53 complex and named MIP (MDM2 Inhibitory Peptide). In addition to the 16-mer peptides, GFP-MIP could bind to TAP-tagged MDM2 immobilized on beads (Fig. 3A). We used MIP for further functional analyses.

### An optimized peptide MIP inhibits the MDM2-p53 interaction

We tested the ability of MIP to inhibit the MDM2-p53 interaction by an ELISA assay that was capable of detecting amounts of MDM2_7–300_ bound to immobilized His_6_-p53 in the presence of different concentrations of synthetic peptides (Fig. 3B). In this assay, MIP could inhibit the interaction with an IC_50_ of 10 nM, which is 29- and 470-fold more potent than the DI and p53_17–28_ peptides, respectively (Table 1). Despite not having selected for binding to MDMX, MIP could also inhibit the MDMX-p53 interaction at an IC_50_ of 120 nM, which was 13- and 250-fold more effective than the DI and p53_17–28_ peptides, respectively (Fig. 3C). These results supported the previous report that the binding interactions of p53 to both MDM2 and MDMX were very similar [26], [27].

**Table 1:** IC_50_ value for MDM2 and MDMX-p53 interaction in ELISA.

Peptides	Sequences	IC_50_ for MDM2 (µM)	IC_50_ for MDMX (µM)
MIP	PRFWEYWLRLME	0.01	0.12
DI	LTFEHYWAQLTS	0.29	1.6
3A	LTAEHYAAQATS	>100	>100
p53_17–28_	QETFSDLWKLLP	4.7	30
MIP (F3A)	PRAWEYWLRLME	0.57	Not tested
MIP (Y6A)	PRFWEAWLRLME	>100	Not tested
MIP (W7A)	PRFWEYALRLME	>100	Not tested
MIP (R9A)	PRFWEYWLALME	0.02	Not tested
MIP (L10A)	PRFWEYWLRAME	1.14	Not tested
MIP (M11A)	PRFWEYWLRLAE	0.4	Not tested

To examine what key residue in MIP is responsible for disrupting the MDM2-p53 interaction, mutational analysis was performed (Table 1). Six residues in MIP (Phe3, Tyr6, Trp7, Arg9, Leu10 and Met11) were replaced by Ala because these six sites were found to be retained in the peptides obtained from the 2nd selection (Fig. 2C). Replacement of Phe3, Tyr6, Trp7, Leu10 and Met11 to Ala lowered the inhibitory ability of the peptide 57-, >10,000-, >10,000-, 114- and 40-fold, respectively. In addition to the known key residues for binding to MDM2 (Phe3, Tyr6, Trp7 and Leu10), Met11 of MIP was identified as a novel key residue. The results suggest that the frequency of the amino acid residues at each position of the peptide enriched from the selection reflects that position's importance in determining affinity to the bait.

### GFP-MIP interacts with MDM2 and activates the p53 pathway in cultured cells

**Figure 4:**
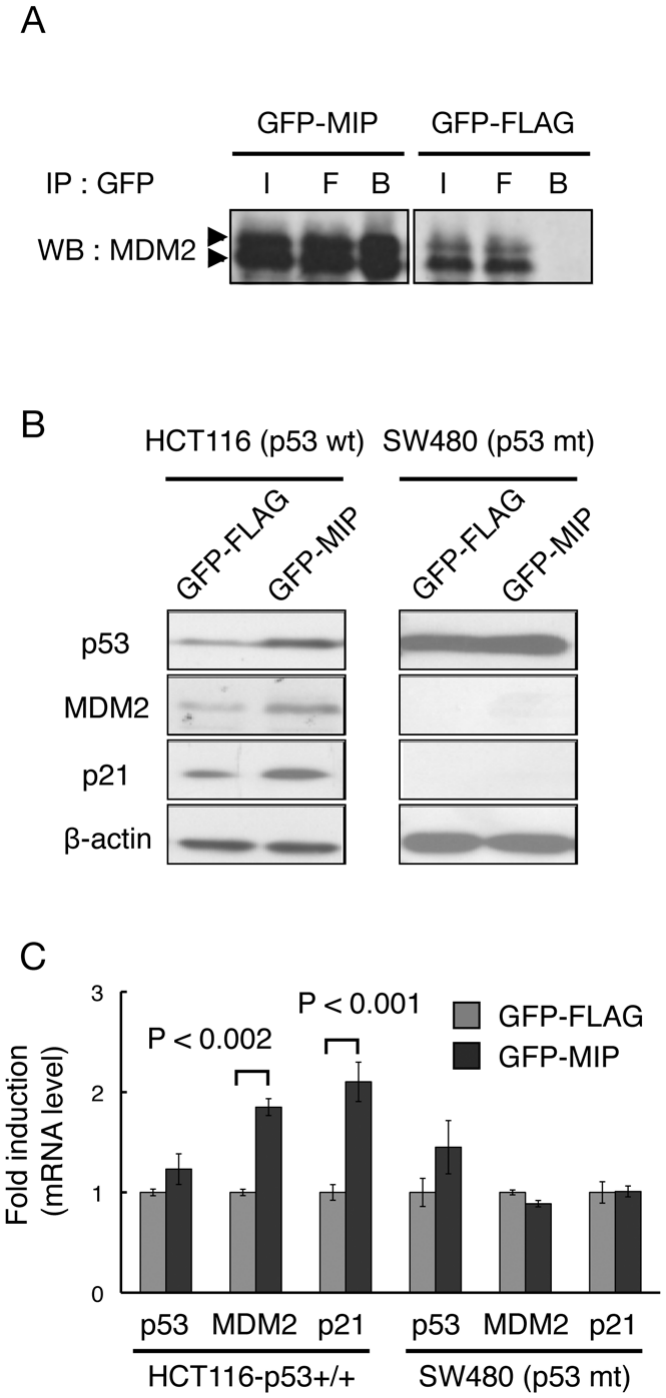
Functional analyses of GFP-MIP in living cells. HCT116-p53+/+ cells or SW480 cells (p53 mt) were transfected with plasmids encoding GFP-fused MIP, GFP-fused FLAG or GFP alone. (A) Immunoprecipitation assays with anti-GFP were performed followed by western blot with an anti-MDM2 antibody. I, input; F, flow-through; B, beads. (B) The whole cell lysates were analyzed by western blot with antibodies against p53, MDM2, p21 and β-actin. (C) mRNA levels of p53, MDM2, p21 and GAPDH were determined by quantitative reverse transcription-PCR using total RNA extracted from the cells. GAPDH was used for normalization.

We tested whether GFP-fused MIP interacted with MDM2 in living cells. In immunoprecipitation assays, MDM2 was coprecipitated with GFP-MIP, but not GFP-FLAG or GFP alone when expressed in human colon carcinoma HCT116-p53+/+ cells (Fig. 4A) expressing wild-type p53. We examined the ability of GFP-MIP to activate the p53 pathway. In contrast to the 16-mer peptides, expression of GFP-MIP caused an increase in p53 and its targets (MDM2 and p21) at the protein level in HCT116-p53+/+ cells but not in SW480 cells containing inactive mutated p53 (Fig. 4B), indicating that the induction of MDM2 and p21 expression was dependent on p53 activity. Moreover, quantitative RT-PCR analysis showed that expression of GFP-MIP increased MDM2 and p21 at the mRNA level in HCT116-p53+/+ cells, whereas no effect on a p53 mRNA level was observed (Fig. 4C), as expected, because the increase in p53 protein level was the result of a decrease in degradation by proteases and not an increase in protein synthesis. However, proliferation of these cells was not inhibited, presumably due to the low transduction or expression efficiency of the plasmid (data not shown).

### Construction of adenoviruses expressing MIP and its functional analyses

To overcome the low transduction efficiency of the plasmid expressing GFP-MIP and for inhibiting tumor cell growth, we prepared a synthetic MIP fused to Tat, a cell-permeable peptide (Tat-MIP). However, the peptide could not activate the p53 pathway (Fig. S2A) but induced necrosis, which is independent of the p53 pathway (Fig. S2B). This result is consistent with previous reports that a p53 peptide fused to a cell-permeable α-helical peptide induced necrosis with cell membrane disruption [28], [29].

**Figure 5:**
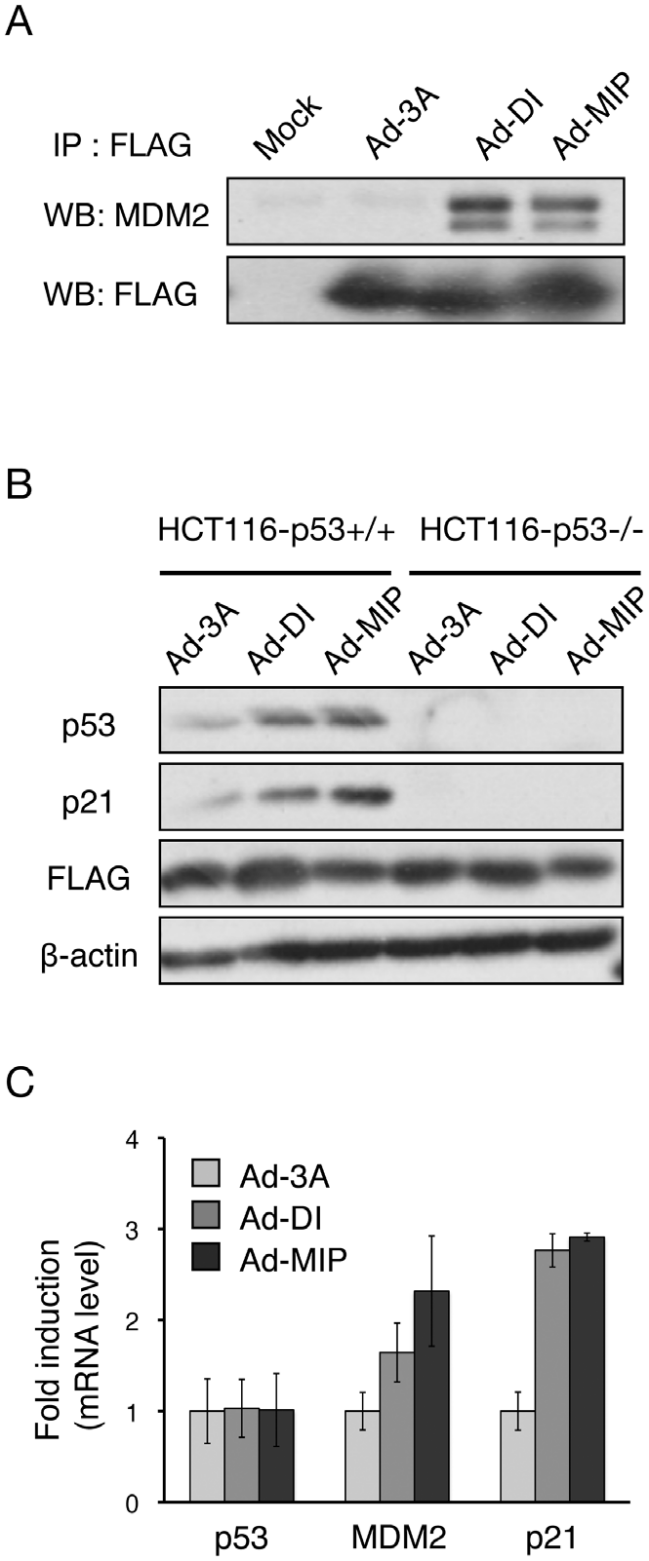
Activation of the p53 pathway by inhibiting the MDM2-p53 interaction. (A) HCT116-p53+/+ cells were infected with 400 MOI of Ad-3A, DI or MIP and 8 MOI of Ad-Cre. After 48 h, immunoprecipitation assay with anti-FLAG antibody was performed followed by western blot with anti-MDM2 or anti-FLAG antibody. (B) HCT116-p53+/+ or HCT116-p53−/− cells were infected with 50 MOI of Ad-3A, DI or MIP and 1 MOI of Ad-Cre. After 24 h, the whole cell lysates were analyzed by western blot with antibodies against p53, p21 and β-actin. (C) HCT116-p53+/+ or HCT116-p53−/− cells were infected with 50 MOI of Ad-3A, DI or MIP and 1 MOI of Ad-Cre. After 24 h, mRNA levels of p53, MDM2, p21 and GAPDH were determined by quantitative reverse transcription-PCR using total RNA extracted from the cells. GAPDH was used for normalization.

We next constructed a recombinant adenovirus expressing MIP fused to a FLAG-tagged thioredoxin scaffold protein (Ad-MIP), as previously described, [30] based on a Cre/loxP adenovirus system. Likewise, DI and 3A (LTAEHYAAQATS; a triple mutant of DI as negative control) expressing adenovirus were constructed (Ad-DI and Ad-3A, respectively). We initially examined the interaction between Ad-MIP and MDM2 by immunoprecipitation assays. The results show that MDM2 coimmunoprecipitated with Ad-MIP or Ad-DI but not with Ad-3A, indicating that Ad-MIP could bind to MDM2 in living cells (Fig. 5A). Furthermore, expression of Ad-MIP increased p53 and its target at the proteins and mRNA level in HCT116-p53+/+ cells but not in HCT116-p53−/− cells (Fig. 5B and C). These results show that Ad-MIP could interact with MDM2 and that the interaction resulted in activation of the p53 pathway similar to GFP-MIP.

**Figure 6:**
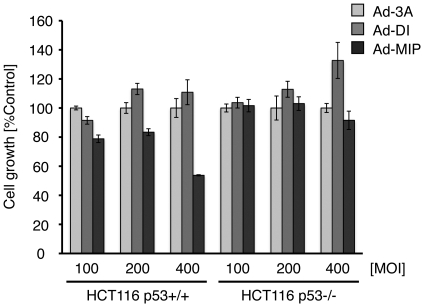
Inhibition of cell growth by Ad-MIP. HCT116 cells p53+/+ or p53−/− were infected with the indicated MOI of Ad-3A, DI or MIP and 1/50 MOI of Ad-Cre. After 72 h, cell viability was analyzed by the WST-1 assay.

We validated the ability of MIP to inhibit tumor cell growth following activation of the p53 pathway. At 400 MOI infection of Ad-MIP, cell growth of HCT116-p53+/+ cells was inhibited to approximately 50% of control, whereas no inhibition of HCT116-p53−/− cells was detected (Fig. 6). In a recombinant adenovirus system we used here, Ad-DI did not inhibit tumor cell growth at titers of up to 400 MOI. These results suggest that Ad-MIP could inhibit tumor cell growth in a p53-dependent manner more potently than Ad-DI.

## Discussion

In the development of anticancer drugs, inhibition of the MDM2-p53 interaction is a very important target. In previous studies, several MDM2-binding peptides, such as DI, that mimic a MDM2 binding domain of p53 have been identified by selection from a randomized library using phage display [12]. Library size in this selection system has been limited to at most ∼10^8^ [31] molecules, whereas the number of possible sequences of 12-mer randomized peptides is 4.1×10^15^. These previously identified peptides were not selected from all of the possible sequences and therefore may not be optimized for binding to MDM2. In this study, we performed selection of MDM2-binding peptides from a random library containing all of the possible sequences by dividing the selection process into two stages to obtain potent peptides. While the library size of mRNA display does not cover 4.1×10^15^ possible sequences, determining key residues that could not be replaced by other residues resulted in a reduction of the number of possible sequences. After determining a key binding motif, the 2nd selection from a partially randomized library led to improvement of subsequent peptides. Furthermore, by repetition of the selection round in a stepwise manner, we were able to eventually identify an optimal peptide with the desired function. Consequently, we identified MIP as an optimized peptide sequence for binding to MDM2 from all possible sequences.

It should be noted that we obtained not only MIP but also peptide X12-2 with approximately the same frequency in the 2nd selection (Fig. 2B). Although *in vitro* binding assay showed that X12-2 binds to MDM2, GFP-X12-2 did not activate p53 pathway in living cells (data not shown). Therefore, we concluded that MIP was more potent than X12-2 and identified MIP as an optimal peptide for inhibiting the MDM2-p53 interaction. Moreover, from the peptide sequences obtained from the 1st selection (Fig. 2A) as well as the previous reports [24], we postulated that three hydrophobic residues corresponding to Phe19, Trp23, and Leu26 of wild-type p53 as the optimal binding motif to bind to MDM2, and we fixed these residues in the 2nd selection. However, peptide X16-9 possessed Leu at the position of Trp23, suggesting that substitution of Trp to Leu is tolerated at the position, and thus the possibility remains that another optimal peptide will be obtained from a library with fixed Leu23.

Recently, Zondlo et al. showed that mutation of Pro27Ser in the p53_17–28_ peptide enhanced its affinity for MDM2 because the mutation increased the α-helical property of the p53_17–28_ peptide [32]. In this study, we found that Met11 of MIP was important for binding to MDM2. Because the position of Met11 of MIP corresponds to Pro27 of p53, the residue might contribute to an increase in the α-helical nature of the peptide. Although we have not yet determined how Met11 in MIP is involved in binding to MDM2, structural analyses of MDM2 complexes with selected peptides in the future will be helpful in determining precisely what key residues in MIP bind to MDM2. In addition, from the structural information, low-molecular-weight compounds that mimic MIP can be designed as in the case of Nutlin-3 [10]. Low-molecular-weight compounds have several advantages over peptide inhibitors, such as high cell permeability and low cost. The strategy based on the three-dimensional conformation of an oncoprotein and a peptide inhibitor complex will lead to the establishment of a high throughput system for developing novel anti-cancer drugs.

Although our aim was not to select peptides for binding to the MDM2 homolog, MDMX, we found that MIP could also inhibit the MDMX-p53 interaction more effectively than DI. A recent study revealed that structures in the p53-binding domains of both MDM2 and MDMX were very similar [27]. The structural study and our results suggest that the binding affinity of the peptides to MDM2 is directly proportional to the affinity of the peptides to MDMX binding. MDMX also binds to p53 and inhibits the anti-tumor activity as well as MDM2 [33]. As inhibiting MDM2 or MDMX leads to significant cancer therapy *in vivo*, targeting both MDM2 and MDMX was effective for optimal p53 activation [33]. Therefore, MDM2/MDMX dual specific inhibitors can be potential cancer therapeutics. However, Nutlin-3 cannot bind to MDMX, most likely due to the presence of various sequence differences in its p53-binding pocket compared to MDM2 [26], [27]. Although Nutlin-3 mimics key residues involved in the binding to MDM2 (Phe19, Trp23 and Leu26 of p53), a recent study showed that Tyr100 in MDM2 accommodates the binding of Nutlin-3, whereas Tyr99 in MDMX causes a steric clash with Nutlin-3 [26]. p14ARF is known as p53 activator by inhibiting MDM2 [34]. Despite previous study that showed that p14ARF also bind to both MDM2 and MDMX, we identified no peptide sequences that were similar to p14ARF, indicating that p53-derived peptides are more suitable for inhibiting MDM2- and MDMX-p53 interaction than that of p14ARF.

In this study, we showed the usefulness of mRNA-displayed peptide selection in multiple stages. Because performing selection in two stages could decrease possible sequences in a randomized peptide library, those of a 12-mer partially randomized library could be easily represented by the library size of mRNA display. In addition, mRNA display has substantial advantages over phage display for peptide selection, in which (i) the library size of the former is much larger than that of the latter, and (ii) cell-free translation of the former is compatible with the incorporation of unnatural amino acids [35]–[38].

## Materials and Methods

### Preparation of bait protein

Oligonucleotides used in this study are presented in Table S1. The cDNA of MDM2 (7–300 amino acids) was amplified by PCR using MDM(1–294)-f and MDM(1–294)-r primers from an A549 cell-derived cDNA library. The PCR product was re-amplified by PCR using 5′adaptorO29T7EcoR and Flag1A-lib primers and cloned into the pDrive cloning vector (Qiagen, Valencia, CA, USA). From the resulting plasmid, the MDM2_7–300_ coding DNA was amplified by PCR using Bam-MDM-f and MDM294-Xho-r primers, digested with BamHI and XhoI, and subcloned into the BamHI/XhoI site of the pCMV-CBPzz vector [39]. The pCMV-CBPzz vector contains a SP6 promoter, a part of the omega sequence named O′ [40], an N-terminal T7-tag coding sequence, and a C-terminal affinity tag, the coding sequence for the IgG binding domain of protein A (ZZ domain), a TEV protease cleavage site and a calmodulin binding peptide [25]. From the resulting pCMV-MDM294-CBPzz plasmid, a bait template DNA was amplified by PCR using SP6-O′-T7 and 3′FosCBPzz primers. The PCR product was purified with the QIAquick PCR purification kit (Qiagen) and used as a template for *in vitro* transcription with a RiboMax large-scale RNA production system-SP6 (Promega, Madison, WI, USA). The RNA was purified with an RNeasy mini kit (Qiagen) and *in vitro*-translated in a wheat germ cell-free translation system (Promega) to produce the MDM2 protein as bait.

### Construction of random mRNA-displayed peptide libraries

The 16-mer random DNA library was amplified from G4SG4S(NNS)16FLAGA6r by PCR using priSP6OGf and priFLAGA6r primers. The 12-mer random DNA library was amplified from X12(FWL)-r using 5′O29-T7-EcoRI and Flag1A-lib primers. The PCR products were purified with the QIAquick PCR purification kit and transcribed into RNA. The resulting RNA was purified with the RNeasy mini kit and ligated with a PEG-Puro spacer [p(dCp)_2_-T(Fluor)p-PEGp-(dCp)_2_-puromycin] [39] using T4 RNA ligase (Takara, Otsu, Japan). The ligated RNA was purified with the RNeasy mini kit and *in vitro*-translated in the wheat germ cell-free translation system to create the mRNA-displayed peptide library [39].

### Preparation of IgG beads

The IgG-Immutex-MAG beads were prepared as follows. Twenty milligrams of Immutex-MAG (MAG2101) (JSR, Tokyo, Japan) were washed three times with PBS containing 0.01% Triton X-100. EDC (0.25 mg/ml) was subsequently added and mixed on a rotator for 90 min at room temperature. Chempure rabbit IgG (Jackson Immunoresearch, West Grove, PA, USA) was then added and mixed on a rotator for 16 h at room temperature. After removal of the supernatant, wash buffer (PBS, 0.1% BSA and 0.01% Triton X-100) was added and incubated for 1 h at room temperature. The beads were subsequently washed five times with wash buffer and stored in PBS containing 0.1% BSA, 0.01% Triton X-100 and 0.02% NaN_3_ at 4°C.

### Affinity selection

The bait protein was added to the IgG-Immutex-MAG beads that had been pre-equilibrated with IPP150 (10 mM Tris-HCl, pH 8.0, 150 mM NaCl and 0.1% NP-40) and mixed on a rotator for 1 h at 4°C. The beads were washed three times with IPP150 prior to the addition of the mRNA-displayed peptide library. The beads/mRNA-displayed peptide library were mixed on a rotator at 4°C for 10 min (5 min after the third round). The beads were washed eight times (13 times after the third round) with IPP150 and twice with TEV cleavage buffer (10 mM Tris-HCl, pH 8.0, 150 mM NaCl, 0.1% NP-40, 1 mM DTT and 5 mM EDTA), and 20 U of TEV protease (Invitrogen, Carlsbad, CA, USA) was added. Rotation was continued for 2 h at 16°C. The resulting eluate was used as the RT-PCR template. RT-PCR was performed with the OneStep RT-PCR kit (Qiagen) using the 5′O29-f and 3′Flag1A primers. The RT-PCR product was used for the next round of selection as described above. After five rounds of affinity selection, the RT-PCR product was cloned using a PCR cloning kit (Qiagen) and sequenced with an ABI PRISM 3100 Genetic Analyzer (Applied Biosystems, Foster, CA, USA).

### Preparation of the GFP-fused peptides

Two oligonucleotides, GFP-fus-MIPf and GFP-fus-MIPr, were phosphorylated with T4 polynucleotide kinase (Takara) followed by ethanol precipitation. The resulting phosphorylated oligonucleotides were annealed by mixing and heating to 98°C for 20 sec and gradually cooled to room temperature. The DNA was cloned into the HindIII/EcoRI site of the pQBI25-HL4 vector [41]. The pQBI25-HL4-FLAG vector used for expressing GFP-FLAG was generated as previously described [42]. The resulting pQBI25-HL4-MIP or pQBI25-HL4-FLAG plasmid was *in vitro*-transcribed and translated in a TNT coupled wheat germ extract system (Promega) to produce GFP-fused peptides.

### *In vitro* binding assay

The GFP-tagged peptide was incubated with MDM2 immobilized on IgG beads for 1 h at 4°C. The beads were washed three times with IPP150, followed by vortexing to elute the bound molecules. The resulting eluate was loaded on a 10% SDS-PAGE gel, and the fluorescence of the GFP tag was detected with a Molecular-Imager FX (BioRad, Richmond, CA, USA).

### ELISA

His_6_-tagged p53 was expressed in *E. coli* as follows: The full length of the p53 gene was cloned into the NdeI/BamHI site of pET15b (Novagen, San Diego, CA, USA). The resulting plasmid was transformed into the *E. coli* strain BL21 (DE3) codon+. The cells were grown in LB with 50 µg/ml carbenicillin at 37°C to an optical density (OD_600_) of 0.5 and induced with 1 mM IPTG for 6 h. Inclusion bodies from the medium were lysed with 50 mM Tris-HCl, pH 8.5, 10 mM DTT and 8 M urea from which the His_6_-p53 was prepared. The His_6_-tagged p53 (2.5 µg/ml PBS) was immobilized on the wells of Hisgrab copper-coated, high-binding-capacity plates (Pierce, Waltham, MA, USA) by incubation at 4°C for 16 h. After washing with PBST, the plates were blocked with 5% skim milk at 4°C for 30 min. *In vitro*-translated MDM2 (7–300 amino acids) mixed with the synthetic peptides MIP, DI, 3A or p53_17–28_ in binding buffer (5% skim milk, 10% glycerol) was added to the wells. The plates were washed after incubating at room temperature for 1 h and were subsequently incubated with antibodies against MDM2 (SMP14, Santa Cruz, Santa Cruz, CA, USA) or FLAG M2 (Sigma, St. Louis, MO, USA) followed by incubation with HRP-labeled anti-mouse IgG (Jackson Immunoresearch). The binding amount of MDM2 or MDMX was measured by the ELISA POD substrate TMB kit (Nacalai Tesque, Kyoto, Japan) with a SAFIRE micro plate reader (Tecan, Männedorf, Switzerland). To test the ability of the peptides to inhibit MDMX-p53 interaction, the cDNA of MDMX (1–200 amino acids) was amplified by PCR using the T7-MDMX(1–200)f primer and the MDMX-(1–200)-FLAG construct from a Human Mosaic cDNA template (Genofi, San Clemente, CA, USA) and re-amplified by PCR using 5′O29-f and MDMX(1–200)-FLAG. The PCR product was purified with the QIAquick PCR purification kit and used as a template for in vitro transcription with the RiboMax large-scale RNA production system-SP6. The RNA was purified with the RNeasy mini kit and *in vitro*-translated in a wheat germ cell-free translation system to produce MDMX protein.

### Cell lines

The tumor cell lines HCT116-p53+/+ and HCT116-p53−/− cells were kindly provided by Dr. Bert Vogelstein (Johns Hopkins University) and were maintained in McCoy's 5A medium with 10% fetal bovine serum and 1% penicillin/streptomycin. SW480 cells purchased from the American Type Culture Collection (ATCC, Manassas, VA, USA) in 2005 and Saos-2 cells purchased from RIKEN Cell Bank (Ibaraki, Japan) in 2007 were maintained in DMEM with 10% fetal bovine serum and 1% penicillin/streptomycin.

### Immunoprecipitation assay

HCT116-p53+/+cells were transfected with pQBI25-HL4-MIP or pQBI25-HL4-FLAG (containing a C-terminal FLAG tag) using the Lipofectamine2000 reagent (Invitrogen). After 24 h, the cells in a 60-mm dish were rinsed once with ice-cold PBS and lysed with 500 µl of TNE buffer (10 mM Tris-HCl, pH 7.8, 150 mM NaCl, 1 mM EDTA, 1% NP-40). The cells in the suspension were separated by centrifugation at 12,000 *g* for 20 min. Twenty microliters of agarose conjugated anti-GFP (Medical and Biological Laboratories, Tokyo, Japan) or anti-FLAG M2 agarose (Sigma) was added to the resulting supernatant and rotated for 2 h at 4°C. The agarose beads were washed five times with TNE buffer and resuspended in SDS-PAGE loading buffer for immunoblot analysis.

### Western blot analysis

Whole cell lysate was analyzed by western blot analysis with antibodies against p53 (Cell signaling, Beverly, MA, Japan), MDM2 (SMP14, Santa Cruz), p21 (SX118, Pharmingen, San Diego, CA, USA) and β-actin (AC-15, Sigma). The blots were developed using an ECL chemiluminescence kit (GE Healthcare, Waukesha, WI, USA).

### Real-time RT-PCR analysis

Total RNA was extracted with the RNeasy mini kit from cells. The RNA was used as template for the real-time RT-PCR reaction with the QuantiTect SYBR Green RT-PCR kit (Qiagen) using the p53F and p53R primer set, the mdm2F and mdm2R primer set or the p21F and p21R primer set. The GAPDH gene was used for normalization with the Light cycler primer sets (Roche, Basel, Switzerland).

### Construction of adenoviruses

DNA fragments encoding three peptides, MIP, DI and 3A, fused to the *E. coli* thioredoxin scaffold protein were prepared as previously described [12]. The recombinant adenoviruses expressing each of the peptides were generated using the adenovirus Cre/loxP kit dual version (Takara). These recombinant adenoviruses were purified by ultracentrifugation on CsCl_2_ gradients and titered using the Adeasy viral titer kit (Stratagene, San Diego, CA, USA). Infection was performed according to the manufacturer's protocol.

### WST-1 assay

Cells (1×10^4^ cells/well) were seeded in a 96-well plate and incubated for 24 h to allow them to attach to the plate. The medium was removed and replaced with fresh medium containing varying MOIs of recombinant adenovirus or different concentrations of synthetic Tat-MIP. The cells were then incubated for 72 h, and the number of viable cells was determined with the cell proliferation reagent WST-1 (Roche) according to the manufacturer's protocol.

## Supporting Information

Figure S1 ***In vitro*** **binding assay of GFP-tagged selected peptides with MDM2-immobilized beads.** GFP-tagged peptides were generated by a transcription/translation reaction and used for the *in vitro* binding assay (see Materials and Methods). I, input; F, flow-through; B, beads.
(TIF)

Figure S2 **Tat-MIP induces necrosis of tumor cells independent of the p53 pathway.** (A) HCT116-p53+/+ cells were treated with DMSO, 10 µM Nutlin-3 or 10 µM synthetic Tat-MIP for 48 h. Whole cell lysates were analyzed by western blot with antibodies against p53, MDM2, p21 and β-actin. (B) HCT116-p53+/+ and HCT116-p53−/− cells were treated with the indicated concentration of synthetic Tat-MIP for 72 h. Cell viability was subsequently analyzed using the WST-1 assay.
(TIF)

Table S1 **Oligonucleotide sequences used in this study.**
(DOC)
