# Supplementary figures and images for: mRNA Display Selection of an Optimized MDM2-Binding Peptide That Potently Inhibits MDM2-p53 Interaction
Source: PLoS One. 2011 Mar 15;6(3):e17898. doi: 10.1371/journal.pone.0017898 (PMC3057987; doi:10.1371/journal.pone.0017898)

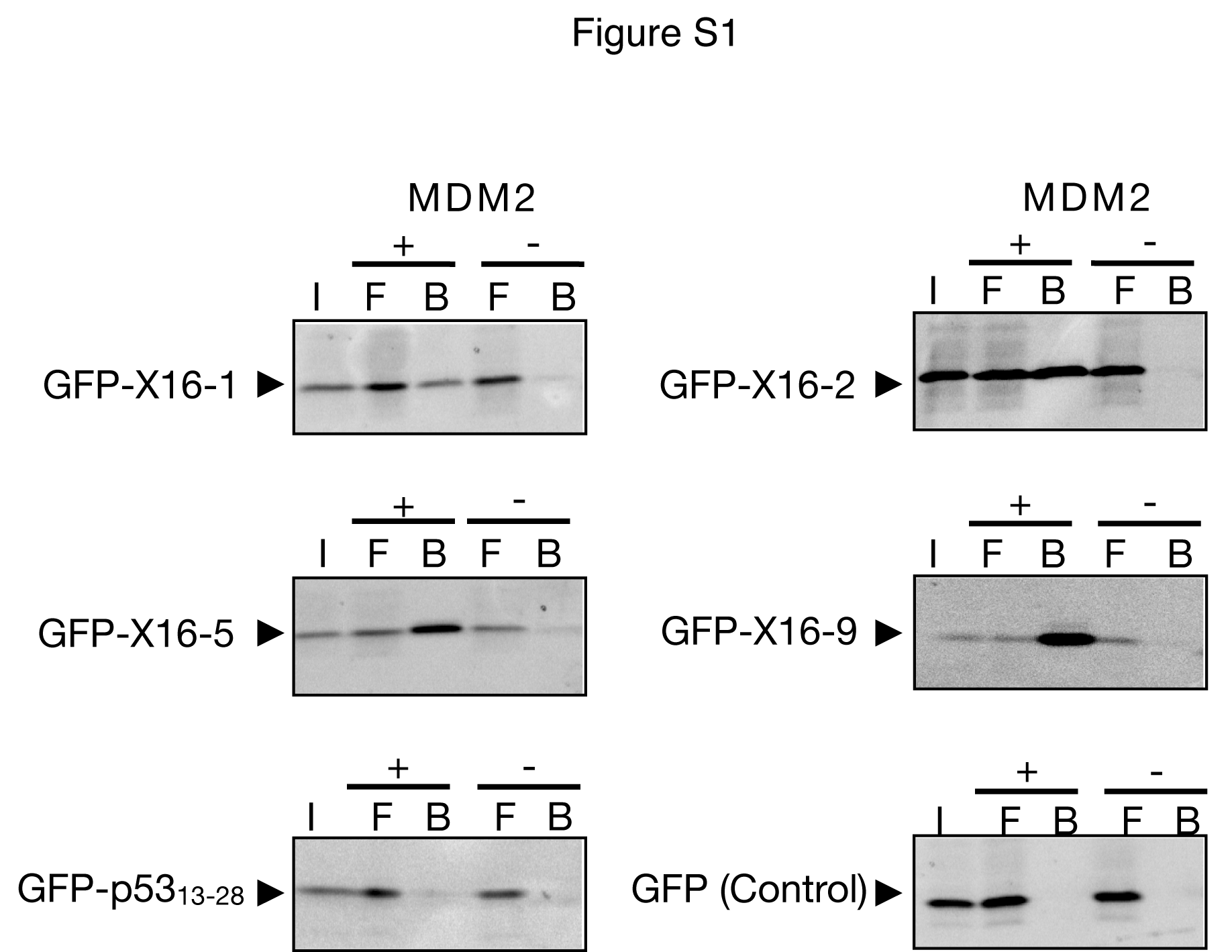

Supplement: Figure S1 — In vitro binding assay of GFP-tagged selected peptides with MDM2-immobilized beads. GFP-tagged peptides were generated by a transcription/translation reaction and used for the in vitro binding assay (see Materials and Methods). I, input; F, flow-through; B, beads. (TIF) [file pone.0017898.s001.tif]

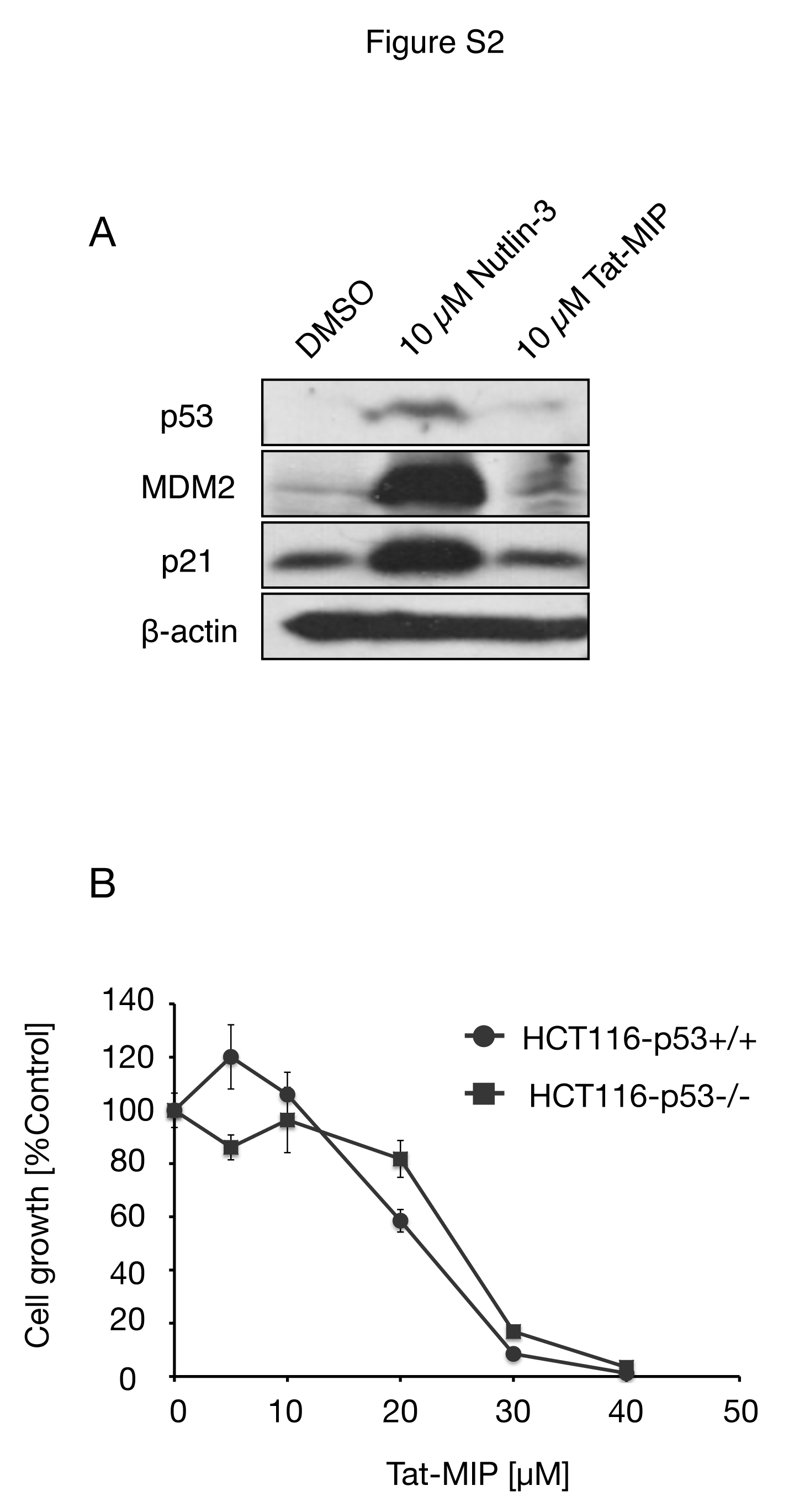

Supplement: Figure S2 — Tat-MIP induces necrosis of tumor cells independent of the p53 pathway. (A) HCT116-p53+/+ cells were treated with DMSO, 10 µM Nutlin-3 or 10 µM synthetic Tat-MIP for 48 h. Whole cell lysates were analyzed by western blot with antibodies against p53, MDM2, p21 and β-actin. (B) HCT116-p53+/+ and HCT116-p53−/− cells were treated with the indicated concentration of synthetic Tat-MIP for 72 h. Cell viability was subsequently analyzed using the WST-1 assay. (TIF) [file pone.0017898.s002.tif]
