# Supplementary material for: mRNA Display Selection of an Optimized MDM2-Binding Peptide That Potently Inhibits MDM2-p53 Interaction
Source: PLoS One. 2011 Mar 15;6(3):e17898. doi: 10.1371/journal.pone.0017898 (PMC3057987; doi:10.1371/journal.pone.0017898)
Supplement: Table S1 — Oligonucleotide sequences used in this study. (DOC) [file pone.0017898.s003.doc]

Table S1. Oligonucleotide sequences used in this study

| Name | Sequence (5’ to 3’) |  |
| --- | --- | --- |
| MDM(1-294)f | CAAATGGCGAATTCCATGTGCAATACCAACATG |  |
| MDM(1-294)r | CTTGTAGTCAAGCTTATCTTCTTCAAATGAATCTGT |  |
| 5'adaptorO29T7EcoR | GAACAACAACAACAACAAACAACAACAAAATGGCTAGCATGACTGGTGGACAGCAAATGGCGAATTCC |  |
| Flag1A-lib | TTTTTTCTTGTCGTCATCGTCCTTGTAGTCAAGC |  |
| Bam-MDM-f | GGTCGCGGATCCATGTGCAATACCAACATGTCTG |  |
| MDM294-Xho-r | CTTGAGCTCGAGATCTTCTTCAAATGAATCTGTATC |  |
| SP6-O’-T7 | GAATTTAGGTGACACTATAGAAACAATTACTATTTACAATTACAATGGCTAGCATGACTGGTGG |  |
| 3’FosCBPzz | GGATCTCCATTCGCCATTCA |  |
| G4SG4S(NNS)16FLAGA6r | TTTTTTCTTATCGTCGTCATCTTTGTAGTC(SNN)_16_TGAGCCTCCGCCTCCTGAACCGCCGCCACC |  |
| priSP6OGf | ATTTAGGTGACACTATAGAACAACAACAACAACAAACAACAACAAAATGGGTGGCGGCGGTT |  |
| priFLAGA6r | TTTTTTCTTATCGTCGTCATCTTTGTAGTC |  |
| X12(FWL)-r | TCGTCATCGTCCTTGTAGTCAAGCTTSNNSNNTAASNNSNNCCASNNSNNSNNGAASNNSNNGGAATTCGCCATTTGCTGTCCACC |  |
| 5'O29-T7-EcoRI | GGAAGATCTATTTAGGTGACACTATAGAACACAACAACAACAAACAACAACAAAATGGCTAGCATGACTGGTGGACAGCAAATGGCGAATTCC |  |
| 5'O29-f | GGAAGATCTATTTAGGTGACACTATAGAACACAACAACAACAAACAACAACAAAATG |  |
| 3'Flag1A | TTTTTTCTTGTCGTCATCGTCCTTGTAGTC |  |
| GFP-fus-MIPf | AGCTGGCGAATTCCCCCAGGTTCTGGGAGTACTGGTTGCGGTTAATGGAGAAGCTTGACTACAAGGACGATGACGACAAGTGAA |  |
| GFP-fus-MIPr | AATTTTCACTTGTCGTCATCGTCCTTGTAGTCAAGCTTCCCATTAACCGCAACCAGTACTCCCAGAACCTGGGGGAATTCGCC |  |
| T7-MDMX(1-200)f | CAACAACAAACAACAACAAAATGGCTAGCATGACTGGTGGACAGCAAATGGCGAATTCCATGACATCATTTTCCACCTCTG | |
| MDMX-(1-200)-FLAG | TTTTTTCTTGTCGTCATCGTCCTTGTAGTCAAGCTTCCAAGGCAGGCCAGCTAC | |
| p53F | CGAGATGTTCCGAGAGCTGA | |
| p53R | TTATGGCGGGAGGTAGACTG | |
| Mdm2F | TAGGAGATTTGTTTGGCGTG | |
| Mdm2R | AGATGTACCTGAGTCCGATGA | |
| p21F | AAGACCATGTGGACCTGT | |
| p21R | TGGAGTGGTAGAAATCTGTC | |
